# Older Patients Are Less Affected by Radiochemotherapeutic Treatment than Younger

**DOI:** 10.1155/2018/5471054

**Published:** 2018-04-15

**Authors:** Max J. Weiling, Wencke Losensky, Katharina Wächter, Teresa Schilling, Fabian Frank, Matthias Haas, Rainer Fietkau, Luitpold V. Distel, Stephanie Weiss

**Affiliations:** ^1^Department of Radiation Oncology, University Hospital Erlangen, Friedrich-Alexander-Universität Erlangen-Nürnberg, Erlangen, Germany; ^2^Department of Radiology, Charité Universitätsmedizin, Berlin, Germany

## Abstract

**Purpose:**

The general assumption is that cancer therapy impairs the quality of life in elderly patients more than in younger ones. We were interested in the effects of radiochemotherapeutic treatment on the quality of life of elderly patients compared to younger patients and compared to normative data of a general German population.

**Methods and Materials:**

A total of 465 patients completed the EORTC QLQ-C30 questionnaire. Repetitive completion of the questionnaire over time led to 1407 datasets. Our patient cohort contained 197 (42.4%) patients with colorectal cancer followed by 109 (23.4%) patients with head and neck cancer, 43 (9.2%) patients with lung cancer, and 116 (25%) with other types of cancer. Patients were categorized into five age groups, the respective cut-offs being 40, 50, 60, and 70 years. Normative data were drawn from a population study of a general German population.

**Results:**

Functional scores and symptom scores were approximately stable between the different age groups. Our data does not suggest a significant difference between the investigated age groups. Advancing age evened out the differences between the normative data of the general German population and the cancer patients in 11 of 15 scores.

**Conclusions:**

The general belief about younger patients having fewer physical and psychological problems related to radiochemotherapy needs to be reconsidered. Overall resilience of older patients is apparently underestimated.

## 1. Background

Over the last years the topic quality of life (QoL) is gaining more and more importance in medical research. Mostly the QoL surveys among cancer patients are used for different groups of cancer patients to improve patient care. Yet, a comparison of patient QoL to the QoL of the general population is challenging. Individuals differ in gender, cultural background, and ethnical and regional origin, to name but a few. Due to physiological changes, it is very important to mention age as a major factor influencing QoL. Nowadays, as the average population ages, elderly patients and their therapies become more important. To yield normative QoL data a representative sample of healthy individuals can be interviewed, for example, the population of a country, and can additionally be classified by age. This kind of study has been conducted for the general population in Germany (GGP) [1–3], Sweden [4], Norway [5], and Colombia [6].

Cancer and cancer therapy have side effects and regularly constrain quality of life. Elderly cancer patients have a high symptom burden and a decreased QoL. It is generally suspected that elderly patients suffer more from cancer and its therapy than younger ones. This is an unsubstantiated belief because there is a lack of studies for elderly people. Age is an exclusion criterion in most studies and therefore only scarce evidence exists for elderly patients. Old patients are at risk of receiving substandard treatment because of the concern of oncologists to generate adverse effects as well as poor functional outcomes and significant decrements in QoL [7]. The term “ageism” is used to describe this practice in cancer therapy discriminating individuals based on their age [8]. Moreover, studies on the impact of old age on generating adverse effects yield contradictory findings [7].

Our aim was to compare the quality of life of a group of cancer patients treated with radiochemotherapy to a group of random adult individuals, reflecting the general German population [1, 2]. We were especially interested in the age specific limitations of functions and appearance of symptoms in the radiochemotherapy treated patients compared to a normative group.

## 2. Material and Methods

### 2.1. Study Population

The study included 465 consecutive patients who agreed to participate by filling in the third version of EORTC QLQ-C30. Patients coming for the first time to our department were informed on the questionnaire based survey. Inclusion criteria to the study were a clinically diagnosed cancer treated by radiotherapy and concomitant chemotherapy (radiochemotherapy). All patients were in the first week of their treatment inpatients. The patient collective is divided into 186 (40%) patients under 60 years and 279 (60%) patients over 60 years. Predominant group were 197 (42.4%) patients with colorectal cancer followed by 109 (23.4%) patients with head and neck cancer and 43 (9.2%) patients with lung cancer. The remaining 116 (25%) individuals were suffering from other types of cancer. Data collection period was between May 2010 and August 2014. Patients having a recurring or metastatic disease were excluded. A total of 465 patients received an individually adapted radiotherapeutic treatment using total doses up to 73 Gy with an average of 54.2 Gy (Table 1). Administered chemotherapeutic treatment was most frequently 5-FU followed by 5-FU combined with Oxaliplatin or Cisplatin. 91 patients died in the follow-up period (Table 1). The Ethics Review Committee of the University Hospital Erlangen approved the study including the use of patients' individual data. All patients gave their written informed consent.

### 2.2. Data Collection

Study participants completed the questionnaire up to six times. A physician or a medical student personally delivered the questionnaire and answered all questions from the patient. The first QLQ-C30 was filled in prior to the patients' first therapy session, the second questionnaire exactly in the middle of the therapy, and the third one or two days prior to the end of therapy. Patients were also asked to participate at their first follow-up examination. To patients who did not appear for the examination, the questionnaire was mailed. The last two questionnaires were sent by letter one and two years after finishing therapy. Questionnaires were digitized using an Excel macro questionnaire. Patients were subdivided into the following age groups: younger than or equal to 39 years; from 40 to 49, 50 to 59, 60 to 69; and 70 years or older. A further division was done by separating patients into an age group younger than 60 years and one of 60 years and older.

### 2.3. EORTC QLQ-C30 Questionnaire

The EORTC QLQ-C30 is a well validated instrument for assessing QoL in cancer patients participating in clinical studies. It is used for almost 30 years and under constant development. Higher values for symptom scores generally indicate higher symptom burden and therefore worse QoL for the patient. In contrast, higher values for functional scores indicate better patient condition. 15 scores, divided into symptom scales and functional scales, were compared. Functional scales are multi-item scales and consisted of either physical, emotional, cognitive, social, or role functioning. Each item is to be answered on a four-point scale, from 1 (not at all) to 4 (very much). The global health status and the quality of life scale each have seven response options ranging from 1 (very poor) to 7 (excellent). Symptom scales were represented by three multi-item scales for fatigue, pain, and nausea/vomiting and six single item scales for diarrhea, dyspnea, insomnia, loss of appetite, constipation, and financial difficulties. According to the manual of the EORTC QLQC30, all scales and items were transformed to a 0–100 range.

### 2.4. General German Population

Radiochemotherapy patients data were compared to the datasets of two general German population surveys termed GGP1 [1, 2] and GGP2 [1, 2]. Aim of the GGP studies is to give normative reference value for a representative German population to better understand and analyze acquired data from patients. GGP1 and GGP2 were conducted in Germany in the year 2012. GGP1 is a representative sample in terms of age, gender, and education from all over Germany including 2448 individuals with a mean age of 50.2 years (range 18 to 92 years). For GGP1 the entire state was separated into 320 sample areas representing different regions. Once a sample area was selected, street, house, and household were selected randomly. All individuals were visited by study assistants. Three attempts were made to contact the selected individuals if they were not at home. GGP2 is a random sample of the population in Lübeck, Northern Germany, including 4684 individuals with a mean age of 51.7 years and minimum age of 16 years. Randomly identified individuals with regard to age, sex, and urban districts were surveyed in spring or summer 2012. For individuals who did not answer the first time, the questionnaire was mailed a second time. Among the GGP2 most common diseases were hypertension (35.7%), hyperlipidemia (25.4%), cardiovascular disease (11.8%), and cancer (8.0%).

### 2.5. Statistics

IBM SPSS Statistics 21 was used for calculations of differences between independent groups using Student's unpaired *t*-test. *p* values < 0.05 were considered significant. Additionally, clinical significance was assumed if an interval of a difference ≥ 10 points was seen between two groups [9].

## 3. Results

### 3.1. Cohort of Cancer Patients

**Figure 1:**
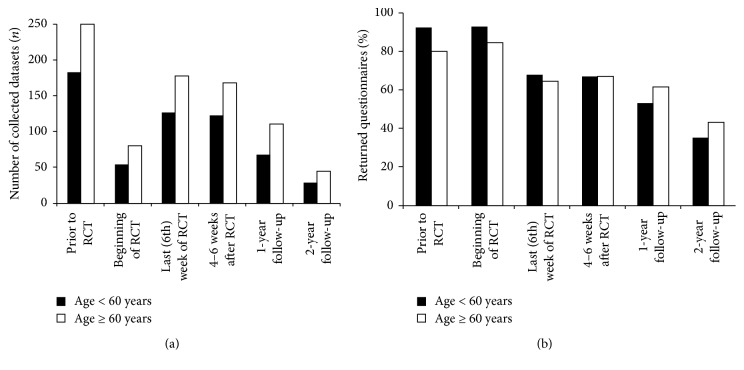
(a) Number of collected datasets at the various times of surveys. (b) Percentage of the returned questionnaires at the various times of surveys.

The survey of 465 patients treated at the University Hospital Erlangen led to a total of 1407 datasets regarding quality of life assessed in the variety of 15 scores. The inclusion criterion was an inpatient stay in the first week of the radiochemotherapeutic treatment. On average, each patient filled in 3.1 questionnaires (Figure 1(a)). Overall 69.5% of all possible surveys have been completed (Figure 1(b)). These scores were divided into functional scores and symptom scores and were analyzed with particular attention to different age categories, focusing on the distinction between the radiochemotherapy patients and the general German population [1]. The largest group of the radiochemotherapy patients, comprising 30.5% of all recruited patients, was the category of individuals being ≥70 years old, followed by 29.5% of all recruited patients in the group of 60- to 69-year-old individuals. The mean age of the cancer patients was 62.3 years with a range from 23.5 to 87.5 years.

### 3.2. Functional Scores of Cancer Patients Compared to the General German Population

**Figure 2:**
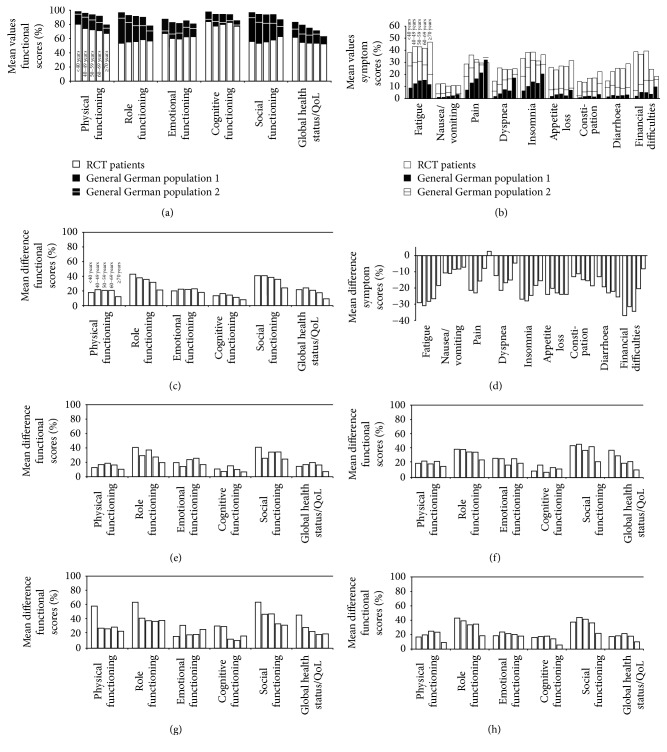
Mean values of all pooled questionnaires (a) functional scores and (b) symptom scores. Radiochemotherapy patients (white bars) are compared to the GGP1 (black bars) and GGP2 (white or black horizontal line). Age is categorized. First bar: <40 years; second bar: 40–49 years; third bar: 50–59 years; fourth bar: 60–69 years; and sixth bar ≥ 70 years. (c) Mean differences between radiochemotherapy patients and GGP1 subdivided by functional scores and symptom scores in dependence of age categories. Functional scores mean differences between radiochemotherapy patients and GGP1 suffering from (e) rectal cancer, (f) head and neck cancer, (g) lung cancer, and (h) all other cancer diagnoses.

Functional scores and symptom scores of all radiochemotherapy patients were pooled and compared to the data originating from Hinz et al., now called GGP1 (Figures 2(a) and 2(b)). Scores of GGP2, the data originating from the second general German population by Waldmann et al., were included in the figures by horizontal lines. Functional scores of the general German population have high levels in all age groups and decrease steadily with age. In contrast, the values of the radiochemotherapy patients are lower and decrease less or even increase slightly from younger to older patients (Figure 2(a)). This leads to a reduction of the difference between the two compared datasets in dependence of age. The maximum deviation (27.5 points) of all functional mean scores occurs in the group of patients being younger than 40 years; the minimum deviation (16.7 points) occurs in the age group ≥ 70 years (Figures 2(a) and 2(c)). The difference between the radiochemotherapy patients and the GGP1 score decreases with advancing age in the scores “physical functioning,” “role functioning,” “cognitive functioning,” and “social functioning,” as well as in “global health status/QoL.” The only exception is “emotional functioning” where the difference remains unchanged at all age categories.

### 3.3. Symptom Scores of Cancer Patients Compared to the General German Population

The symptom scores on the other hand have an opposing trend. The GGP1 has lower symptom levels in all age groups and the average score increases as patients get older. This leads to a diminution of the spread between GGP1 and radiochemotherapy patients. The maximum divergence of 13 points and the minimum divergence of 8 points arise in the same age categories corresponding to the functional scores (Figures 2(b) and 2(d)). The difference between the two compared datasets of the following symptom scores decreases with age: “fatigue,” “nausea/vomiting,” “dyspnea,” “pain,” “insomnia,” and “financial difficulties.” Differences remain the same for “appetite loss” independently of age, while they increase for “constipation” and “diarrhea” the older the patients grow.

### 3.4. Older Patients Compared to Younger

Dividing the sample in younger (<60 years) and older (≥60 years) patients, the differences between both groups for all functional scores, except emotional functioning, are distinctly different (*p* ≤ 0.002). Among the symptom scores, except appetite loss, differences also exist (*p* < 0.001).

### 3.5. Cancer Subgroups

In addition, the difference between GGP1 and the subgroups of colorectal cancer, head and neck cancer, lung cancer, and all other cancer cases was analyzed (Figures 2(e)–2(h), S1–4). Overall there were remarkably little differences between the cancer groups. The trend towards fewer differences between radiochemotherapy patients and GGP1 at older age compared to younger ages was true in all cancer groups. The GGP2 [5] has somewhat worse functional and symptom scores, therefore resulting in considerable differences between the two GGPs (Figure S5, Table S1). However, this decline of disparity also applies for the GGP2 (Figure S5), women and men (Figures S6 and S7), and the four inquiry periods (Figures S8–S11).

### 3.6. Radiotherapeutic Dose and Chemotherapy in the Age Groups

**Figure 3:**
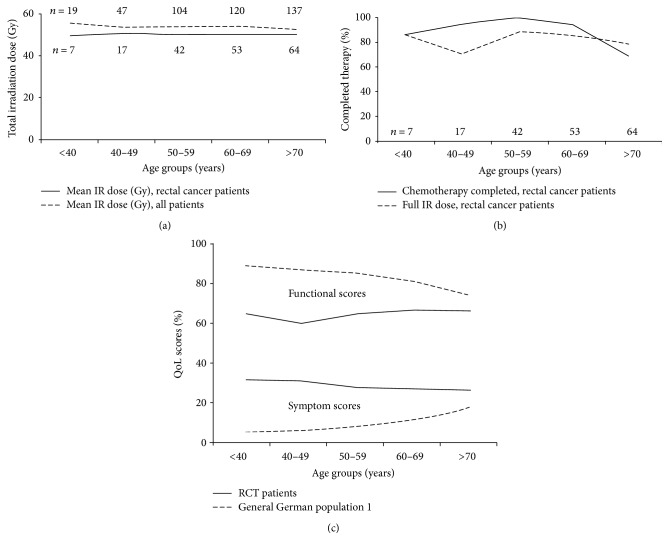
(a) Mean ionizing radiation dose in all patients (stripped line) and rectal cancer patients (solid line). (b) Percentage of patients with completed chemotherapeutic dose in all patients (stripped line) and rectal cancer patients (solid line). (c) Variation of all pooled functional and symptom scores in dependence of age. Solid line: radiochemotherapy (RCT) patients, stripped line: general German population 1.

We also examined the influence of radiation dose for all study participants. The mean value of total therapeutic radiation dose changes only slightly among the age categories (Figure 3(a)). Separating all survey participants into two age groups of patients, < 60 years and ≥60 years, the mean values of both groups differ only slightly by 54.3 Gy and 53.3 Gy (*p* ≥ 0.267; Table 1). Radiotherapy treatment guidelines for rectal cancer patients recommend an overall dose of 50.4 Gy [10]. In the rectal cancer patient group, mean values of total dose among the patients under 60 years (50.3 Gy) and ≥60 years (50.1 Gy) differed only marginally (*p* ≥ 0.5) (Table 2). All patients in the survey received chemotherapy. In the older age group, more cycles of chemotherapy were discontinued before completion or chemotherapeutic dose was reduced (Table 3). 97% of patients being younger than 60 years completed chemotherapy, whereas only 80% of the older age group (≥60 years) received full dose (Figure 3(b), Table 3).

By averaging all the functional or symptom scores of the different age groups and comparing the radiochemotherapy patients to GGP1, in all age groups radiochemotherapy patients differ clinically significantly from GGP1 (Figure 3(c)). The decline of the functional score in the GGP1 with increasing age is obvious, while among the radiochemotherapy patients a trend towards an increase can be detected. The symptom scores increase clearly with age in the GGP1, while among the radiochemotherapy patients even a trend to a decrease of symptoms is observed.

## 4. Discussion

In our study, we investigated whether the age of cancer patients treated with radiochemotherapy was an influencing factor on the quality of life. We could show that younger patients are much more affected by cancer and radiochemotherapeutic treatment compared to older patients. radiochemotherapy patients' QoL was compared to the normative studies of the general German population. There are three studies investigating the QoL of German citizens [1–3]. The apparently healthy individuals of both studies are cross-sectional studies including sick individuals. In the GGP2 11.8% of individuals had cardiovascular disease and 8.0% cancer disease. The trends of a decrease in functions and an increase in symptoms with age were in both GGPs quite similar. The GGP2 has overall somewhat worse score values compared to the GGP1.

With increasing age, the average scores of the GGP1 decrease in functional scores and improve in symptom scores. In contrast, in the group of radiochemotherapeutic patients changes were less pronounced or the scores remained even stable. Thus, we could show that with increasing age the differences between radiochemotherapy patients and GGP1 decrease.

A study in a cohort of 89 cancer patients by Turaka et al. examining QoL during and after radiotherapy showed contrary results. They found that during radiochemotherapy the QoL in patients older than 60 years was lower than in younger patients on a physical, cognitive, emotional, and functional level [11]. Cataldo et al. described similar results in 2013 by demonstrating that with increased age significantly lower rates of symptoms like vomiting or pain occurred [7]. Similar results were also described by Kroenke et al. with younger woman experiencing greater functional losses than middle-aged women [12]. With the lower spread of the symptom scores older patients experience less additional adverse effects than younger patients.

Current data on different populations regard a decline of physical functions and an increase of medical conditions with age as normal. It is generally held true that elderly people have a higher symptom burden than younger people [13]. Therefore older patients may suffer a more pronounced functional impairment and be more vulnerable to the secondary effects of a cancer treatment [14]. Our analysis indicates that these assumptions might be wrong. In contrast to other studies [11], this study shows in almost all evaluated scores that the younger the patients are, the more they have functional impairment. Additionally, their symptom burden rises in several scores. Previous studies described that elderly patients may have achieved an internal reconceptualization of their symptom experience based on their lifetime experiences. They might see especially pain as a normal part of aging and therefore tend to underrate it [15]. Kroenke et al. saw reasons for the higher symptom burden of younger breast cancer women in them having fewer adaptive coping skills, having faced fewer life issues. They grew up in a different historic and social context than middle-aged and older women [12, 16–21].

It is generally believed that older patients receive substandard treatment due to a concern about more severe adverse effects from high doses of radiation and chemotherapy [7]. In this study, we could show that rectal cancer patients of older age received the same therapeutic dose of ionizing radiation (Table 2) as younger patients. Mean radiotherapeutic doses are very similar within the five age groups with a minimal trend to higher dose for younger patients. Chemotherapeutic data were gathered for patients suffering from rectal cancer. Data revealed that the older the patients were, the more chemotherapy was discontinued prematurely or dose was reduced. The question is why chemotherapy was discontinued although the QoL data indicates that patients do not suffer more from therapy than younger patients. To discontinue or reduce chemotherapy that early could be a result of the assumption that older people should rather have no severe adverse effects than finishing their planned therapy. However, it seems that both a completed chemotherapy and no impairment of the patients seem to be feasible.

## 5. Conclusion

This study points out that with increasing age the differences between the cancer patients group and the GGP1 are declining. Older patients have neither more adverse effects than younger patients nor less adverse effects. The general belief about younger patients having fewer problems with both physical and psychological dreadful situation needs to be reconsidered. Possibilities in coping of older patients are obviously underestimated. In conclusion, the treatment of the elderly should be adapted, so that therapy should not be prematurely discontinued or dose reduced solely due to advanced patient age.

## Figures and Tables

**Table 1:** Mean radiotherapeutic total doses of all patients related to age groups and the deceased patients.

Age (years)	*n* (%)	Deceased patients (%)	Mean irradiation dose (Gy)
<40	19 (4.1%)	2 (10.5%)	55.2
40–49	56 (12.0%)	6 (10.7%)	53.7
50–59	111 (23.9%)	13 (11.7%)	54.3
60–69	137 (29.5%)	26 (19.0%)	54.0
≥70	142 (30.5%)	44 (31.0%)	52.6
Total dose			53.7
<60	186 (40.0%)	21 (11.3%)	54.3
≥60	279 (60.0%)	70 (25.1%)	53.5

**Table 2:** Radiotherapeutic characteristics of rectal cancer patients related to age groups.

Age category (years)	Rectal cancer patients (*n*)	Mean IR dose (Gy) (SD)	Completed IR dose (*n*) (%)	Reduced IR dose (*n*) (%)	Increased IR dose (*n*) (%)
<40	7	49.6 (1.9)	6 (85.7)	1 (14.3)	0 (0)
40–49	17	50.8 (4.3)	14 (70.6)	2 (11.8)	1 (5.9)
50–59	42	50.3 (1.9)	37 (88.1)	3 (7.1)	2 (4.8)
60–69	53	50.3 (2)	45 (84.9)	4 (7.5)	4 (7.5)
≥70	64	49.8 (2.9)	52 (78.1)	8 (12.5)	4 (6.3)
Total	183	50.2 (2.6)	150 (82)	18 (9.8)	11 (6)
<60 years	66	50.3 (2.7)	57 (83.3)	6 (9.1)	3 (4.5)
≥60 years	117	50.1 (2.5)	97 (81.2)	12 (10.3)	8 (6.8)

**Table 3:** Chemotherapeutic characteristics of rectal cancer patients related to age groups.

Age category (years)	Rectal cancer patients (*n*)	Chemotherapy completed (*n*) (%)	Reduced dose (*n*) (%)	Standard chemotherapy with 5FU/Oxaliplatin (*n*) (%)
<40	7	6 (85.7)	1 (14.3)	7 (100)
40–49	17	17 (94.1)	0 (0)	12 (70.6)
50–59	42	42 (100)	0 (0)	35 (83.3)
60–69	53	52 (94.3)	1 (1.9)	45 (84.9)
≥70	64	51 (68.8)	13 (20.3)	38 (59.4)
Total	183	168 (86.3)	15 (8.2)	137 (74.9)
<60 years	66	65 (97)	1 (1.5)	54 (81.8)
≥60 years	117	103 (80.3)	14 (12)	83 (70.9)

## Data Availability

The datasets analyzed during the current study are available from the corresponding author on reasonable request.

## Abbreviations

EORTC: European Organisation for Research and Treatment of Cancer
GGP: General German population
QLQ: Quality of Life Questionnaire
QoL: Quality of life.

## Supplementary Materials

Supplementary Materials Additional figures comparing different subgroups to the general population. Supplementary Figure 1: mean values of RCT (radiochemotherapy) patients with rectal cancer compared to the GGP1 (a) functional scores and (b) symptom scores. Supplementary Figure 2: mean values of RCT patients with head and neck cancer compared to the GGP1; functional scores (a), symptom scores (b). Supplementary Figure 3: mean values of RCT patients with lung cancer compared to the GGP1; functional scores (a), symptom scores (b). Supplementary Figure 4: mean values of RCT patients with all other cancer diagnoses compared to the GGP1; functional scores (a), symptom scores (b). Supplementary Figure 5: mean values of RCT patients compared to the second GGP2 (Waldmann) functional scores (a), symptom scores (b), differences of functional scores (c), and differences of symptom scores (d). Supplementary Figure 6: the proportion of men of the RCT patients compared to men of GGP1; functional scores (a), symptom scores (b), differences of functional scores (c), and differences of symptom scores (d). Supplementary Figure 7: the proportion of women of the RCT patients compared to men of GGP1; functional scores (a), symptom scores (b), differences of functional scores (c), and differences of symptom scores (d). Supplementary Figure 8: RCT patients compared to the GGP1 surveyed at the time before RCT; functional scores (a), symptom scores (b), differences of functional scores (c), and differences of symptom scores (d). Supplementary Figure 9: RCT patients compared to the GGP1 surveyed at the time of the last week of RCT (6th week); functional scores (a), symptom scores (b), differences functional scores (c), and differences symptom scores (d). Supplementary Figure 10: RCT patients compared to the GGP1 surveyed at the time of 4–6 weeks after the end of RCT; functional scores (a), symptom scores (b), differences functional scores (c), and differences symptom scores (d). Supplementary Figure 11: RCT patients compared to the GGP1 surveyed at the time of the 1-year follow-up; functional scores (a), symptom scores (b), differences functional scores (c), and differences symptom scores (d). Supplementary Table 1: difference in percent points between GGP and various subgroups for all functional and symptom scores. First row shows differences to GGP2, second to all male patients, third to all female patients, and the other four to our four different inquiry periods.
