# Supplementary material for: Older Patients Are Less Affected by Radiochemotherapeutic Treatment than Younger
Source: Biomed Res Int. 2018 Apr 15;2018:5471054. doi: 10.1155/2018/5471054 (PMC5925006; doi:10.1155/2018/5471054)
Supplement: Supplementary Materials — Additional figures comparing different subgroups to the general population. Supplementary Figure 1: mean values of RCT (radiochemotherapy) patients with rectal cancer compared to the GGP1 (a) functional scores and (b) symptom scores. Supplementary Figure 2: mean values of RCT patients with head and neck cancer compared to the GGP1; functional scores (a), symptom scores (b). Supplementary Figure 3: mean values of RCT patients with lung cancer compared to the GGP1; functional scores (a), symptom scores (b). Supplementary Figure 4: mean values of RCT patients with all other cancer diagnoses compared to the GGP1; functional scores (a), symptom scores (b). Supplementary Figure 5: mean values of RCT patients compared to the second GGP2 (Waldmann) functional scores (a), symptom scores (b), differences of functional scores (c), and differences of symptom scores (d). Supplementary Figure 6: the proportion of men of the RCT patients compared to men of GGP1; functional scores (a), symptom scores (b), differences of functional scores (c), and differences of symptom scores (d). Supplementary Figure 7: the proportion of women of the RCT patients compared to men of GGP1; functional scores (a), symptom scores (b), differences of functional scores (c), and differences of symptom scores (d). Supplementary Figure 8: RCT patients compared to the GGP1 surveyed at the time before RCT; functional scores (a), symptom scores (b), differences of functional scores (c), and differences of symptom scores (d). Supplementary Figure 9: RCT patients compared to the GGP1 surveyed at the time of the last week of RCT (6th week); functional scores (a), symptom scores (b), differences functional scores (c), and differences symptom scores (d). Supplementary Figure 10: RCT patients compared to the GGP1 surveyed at the time of 4–6 weeks after the end of RCT; functional scores (a), symptom scores (b), differences functional scores (c), and differences symptom scores (d). Supplementary Figure 11: RCT patient [file 5471054.f1.pdf]

Supplementary Data

Supplementary figure 1: RCT-patients with rectal cancer compared to the GGP1; functional scores (a), symptom scores (b).

(a)

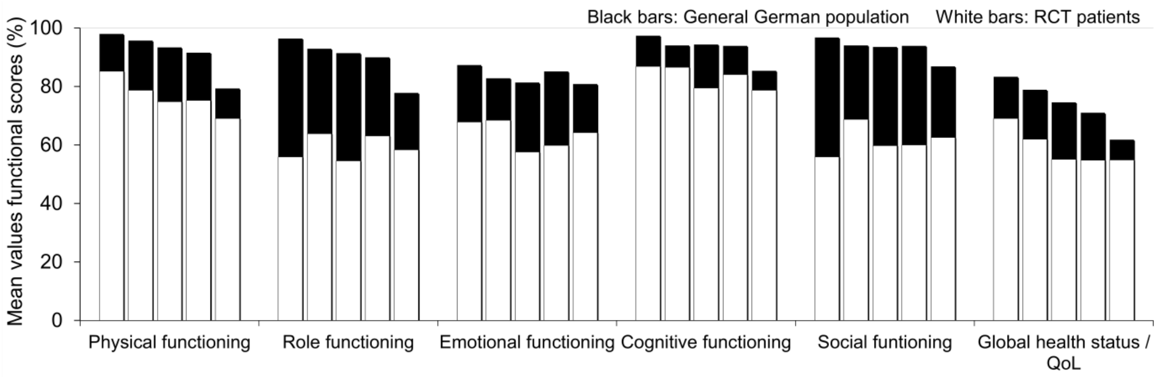

(b)

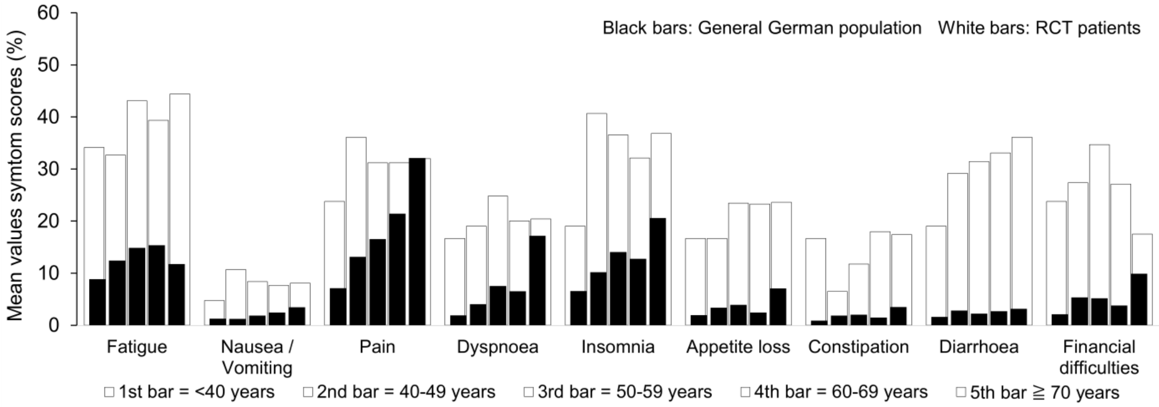

Supplementary figure 2: RCT-patients with head and neck cancer compared to the GGP1; functional scores (a), symptom scores (b)

(a)

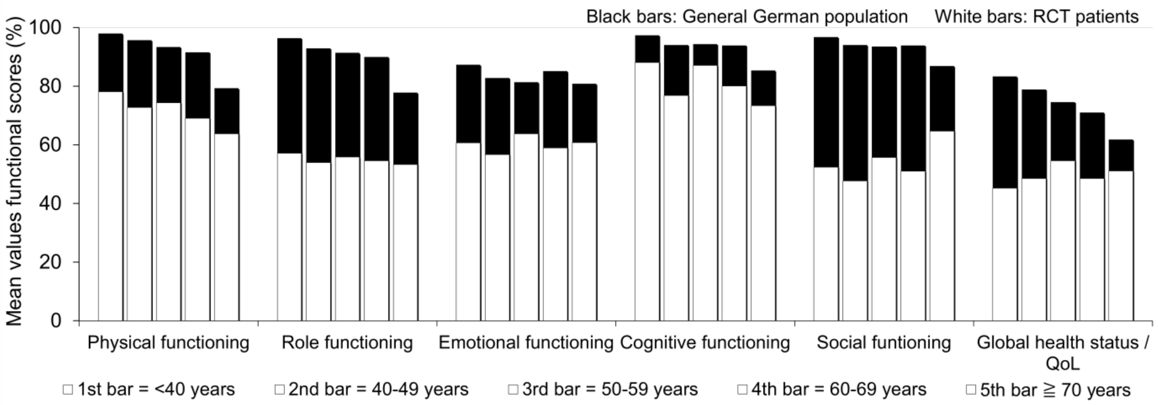

(b)

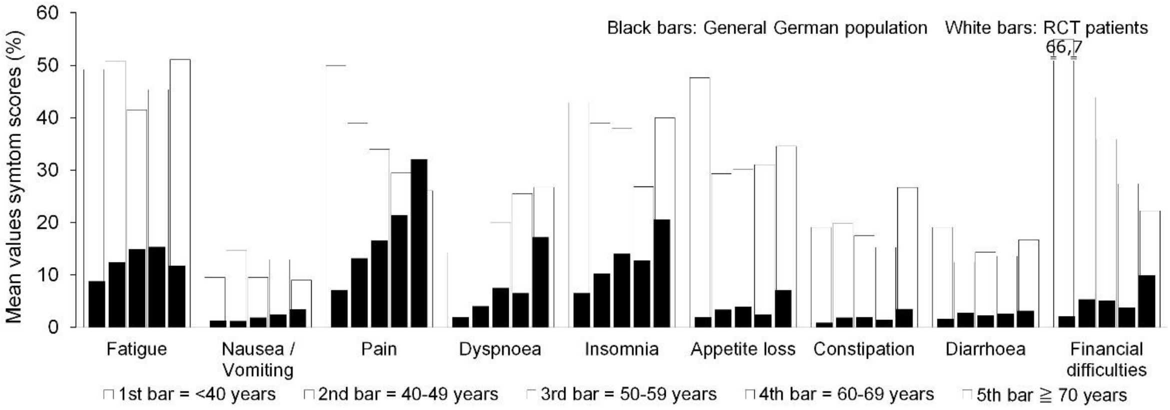

Supplementary figure 3: RCT-patients with lung cancer compared to the GGP1; functional scores (a), symptom scores (b).

(a)

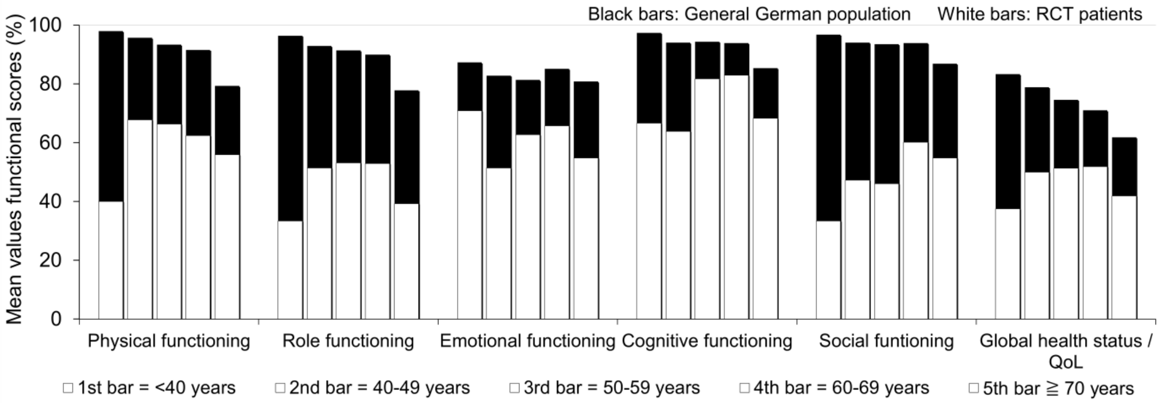

(b)

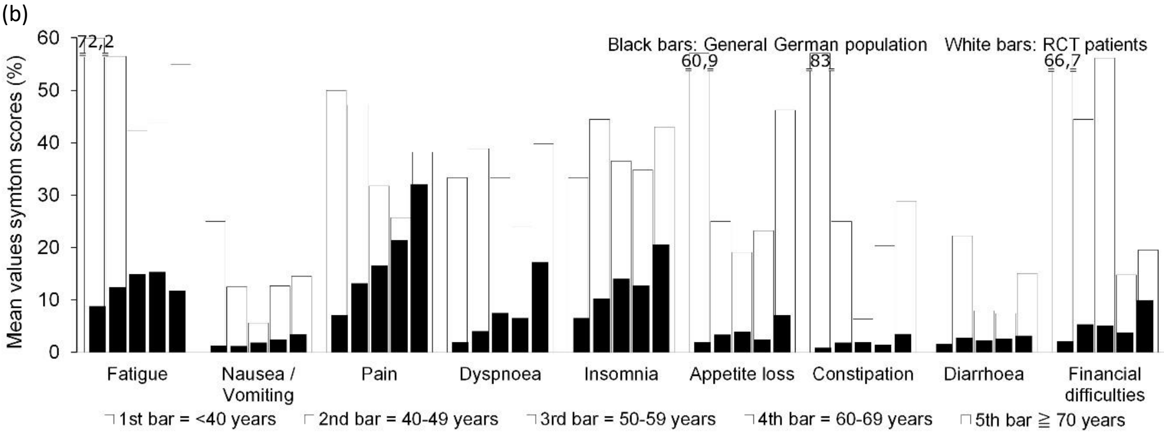

Supplementary figure 4: RCT-patients with all other cancer diagnoses compared to the GGP1; functional scores (a), symptom scores (b).

(a)

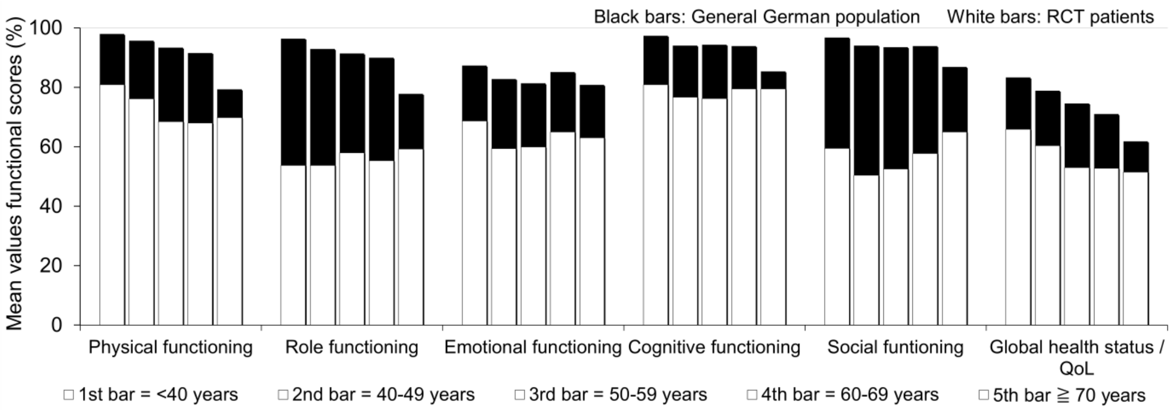

(b)

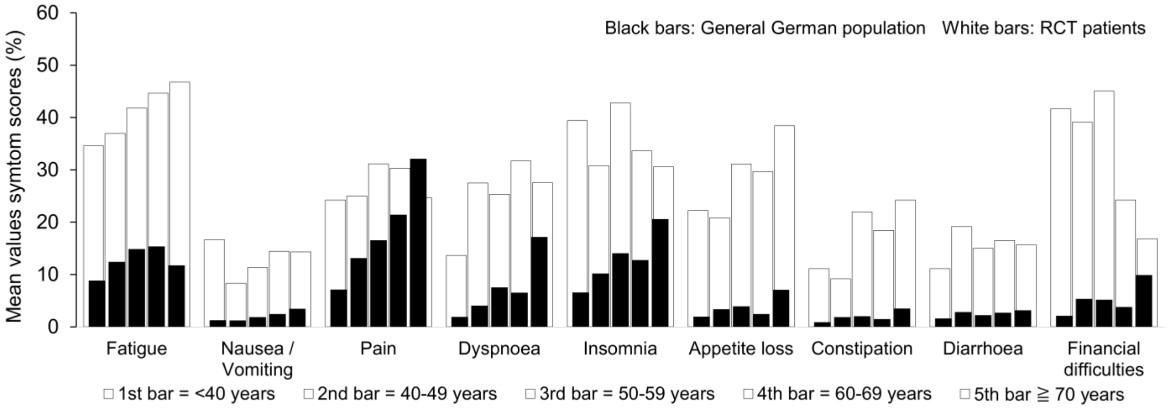

Supplementary figure 5: RCT-patients compared to the second GGP2 (Waldmann) functional scores (a), symptom scores (b), differences functional scores (c), differences symptom scores (d)

(a)

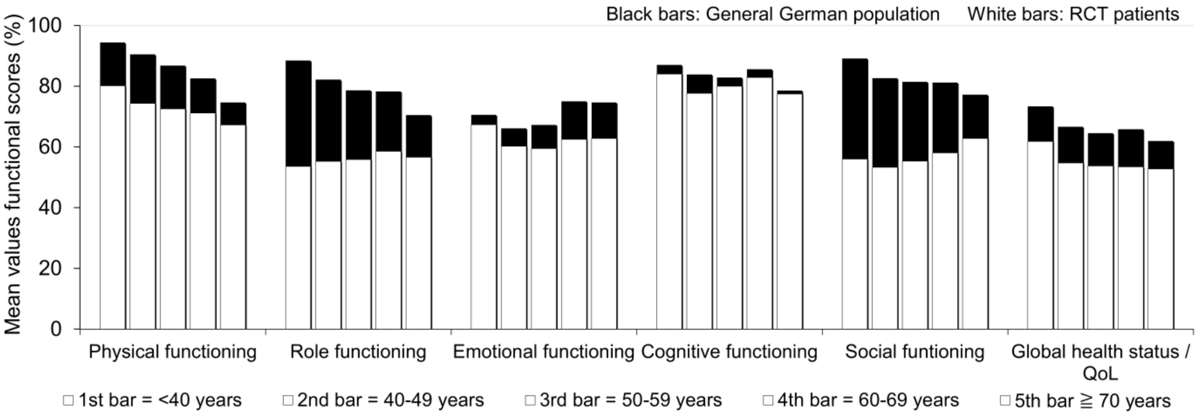

(b)

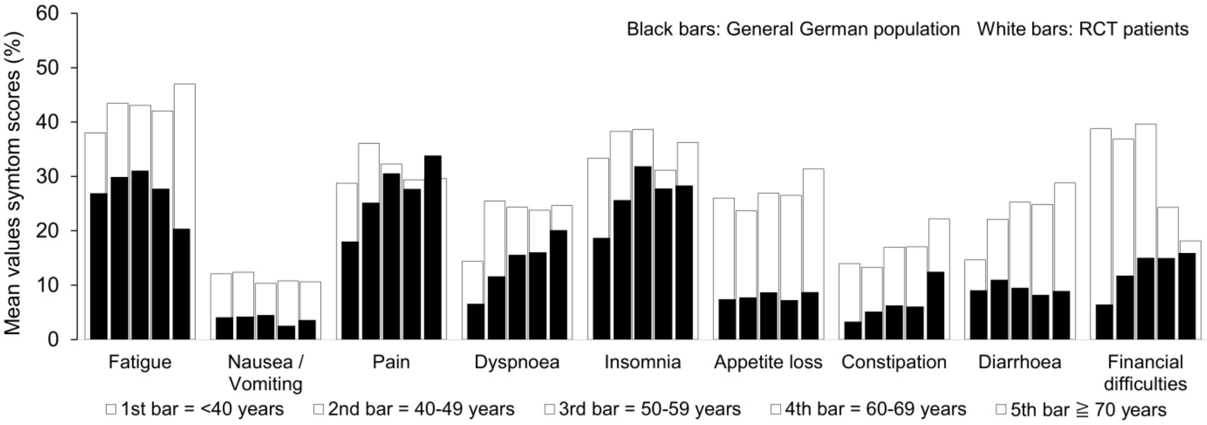

(c)

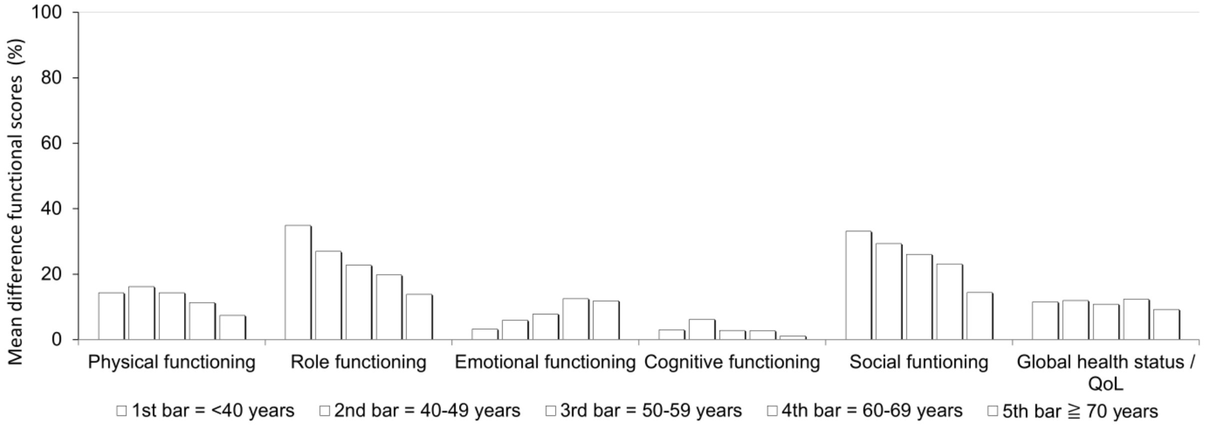

(d)

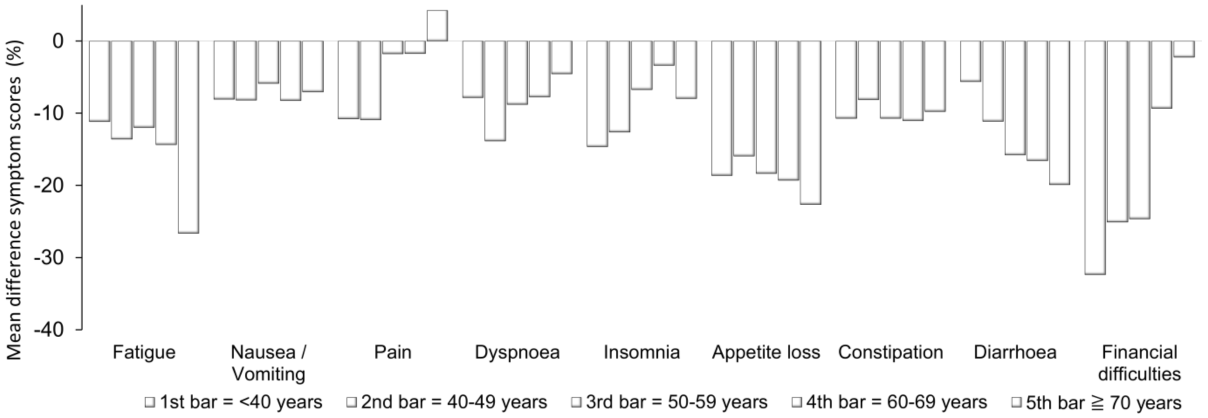
Mean difference symptom scores (%)
0
-10
-20
-30
-40
Fatigue
Nausea / Vomiting
Pain
Dyspnoea
Insomnia
Appetite loss
Constipation
Diarrhoea
Financial difficulties
1st bar = <40 years
2nd bar = 40-49 years
3rd bar = 50-59 years
4th bar = 60-69 years
5th bar ≧ 70 years

Supplementary figure 6: The proportion of men of the RCT-patients compared to men of GGP1; functional scores (a), symptom scores (b), differences functional scores (c), differences symptom scores (d)

(a)

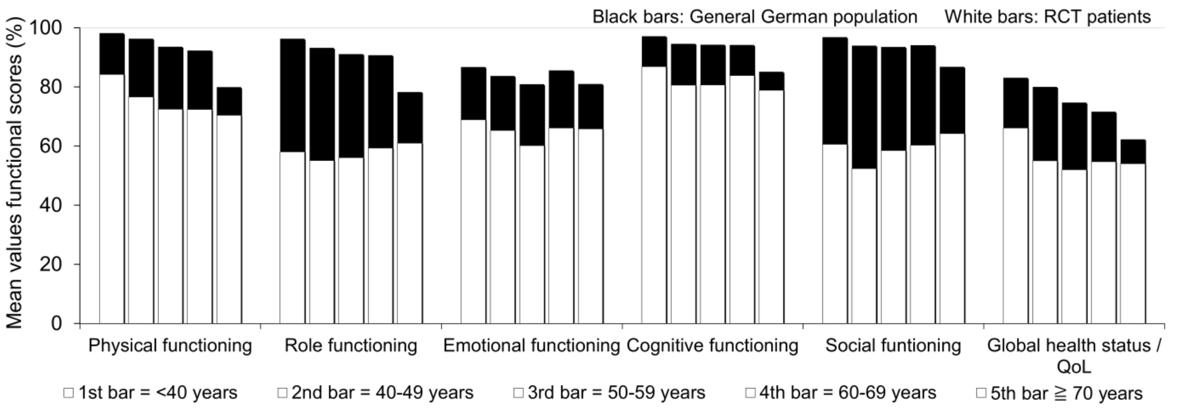

(b)

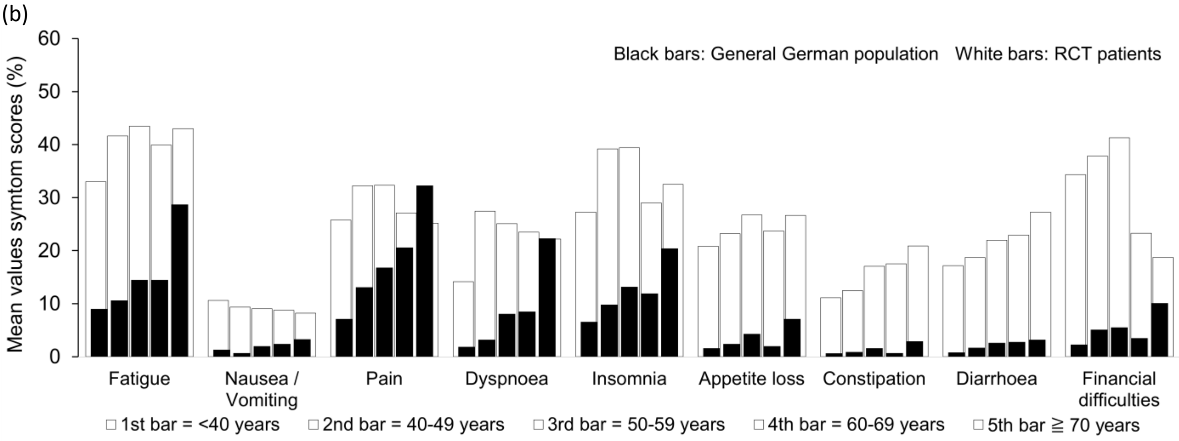

(c)

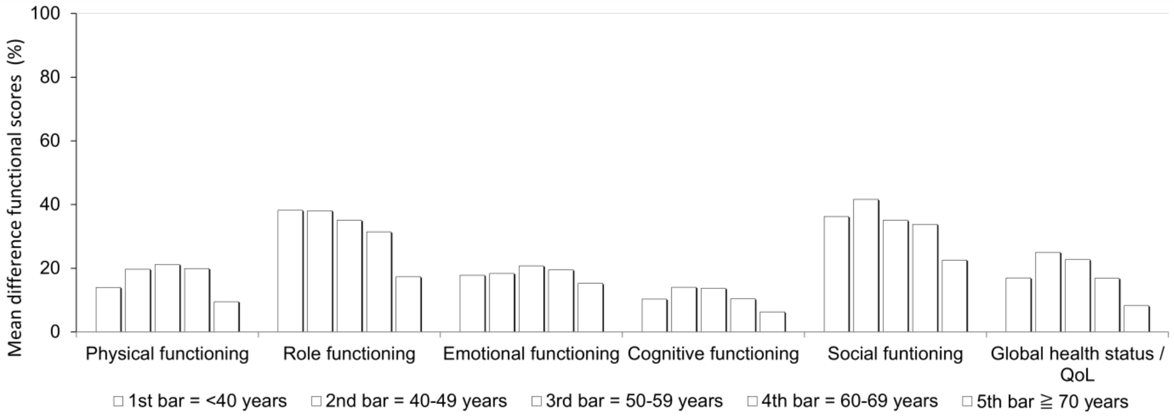

(d)

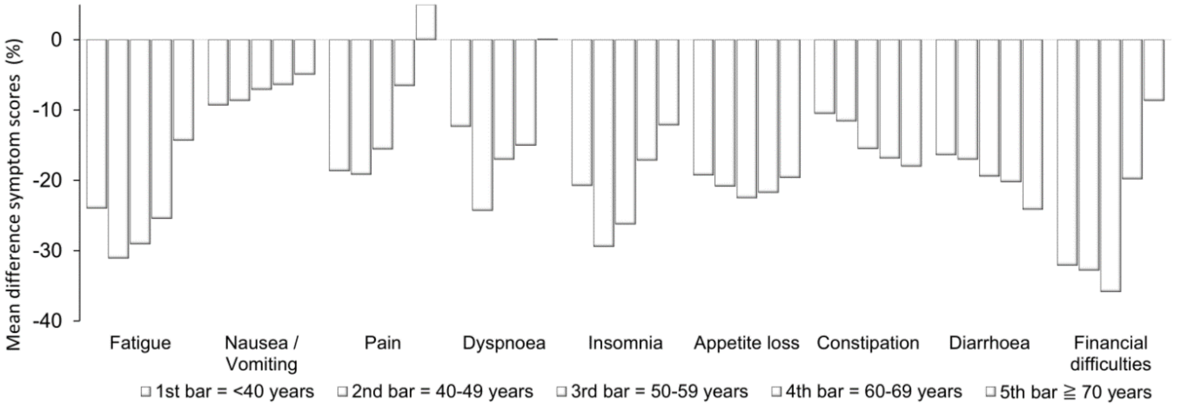
0
-10
-20
-30
-40
Mean difference symptom scores (%)
Fatigue
Nausea / Vomiting
Pain
Dyspnoea
Insomnia
Appetite loss
Constipation
Diarrhoea
Financial difficulties
1st bar = <40 years
2nd bar = 40-49 years
3rd bar = 50-59 years
4th bar = 60-69 years
5th bar ≧ 70 years

Supplementary figure 7: The proportion of women of the RCT-patients compared to women of GGP1; functional scores (a), symptom scores (b), differences functional scores (c), differences symptom scores (d)

(a)

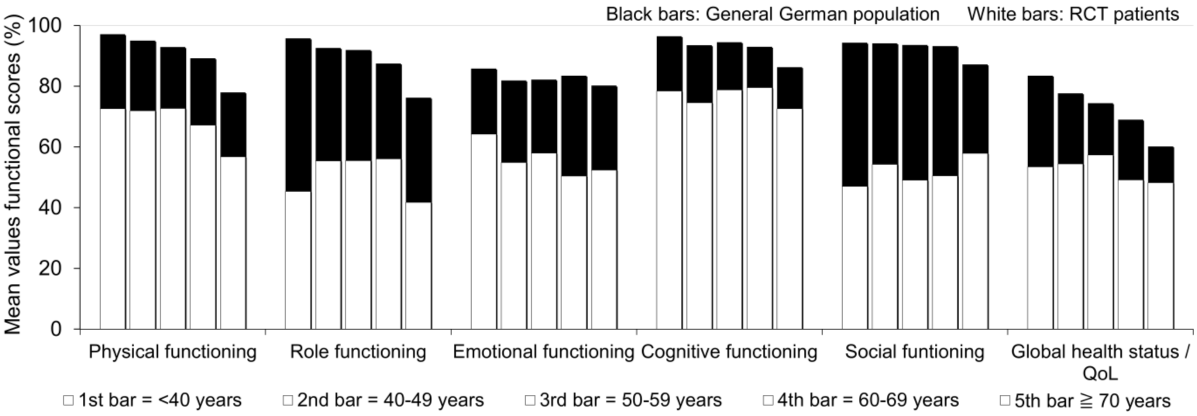

(b)

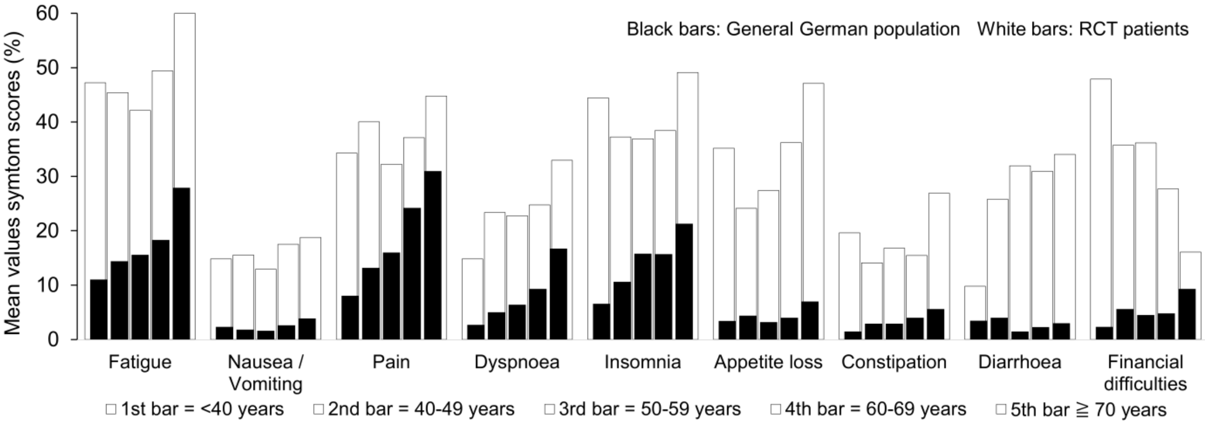

(c)

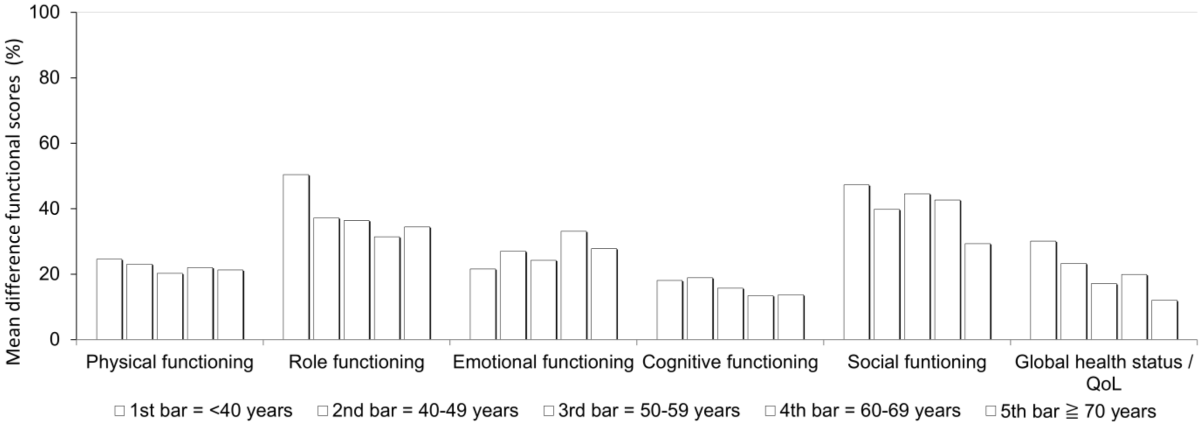

(d)

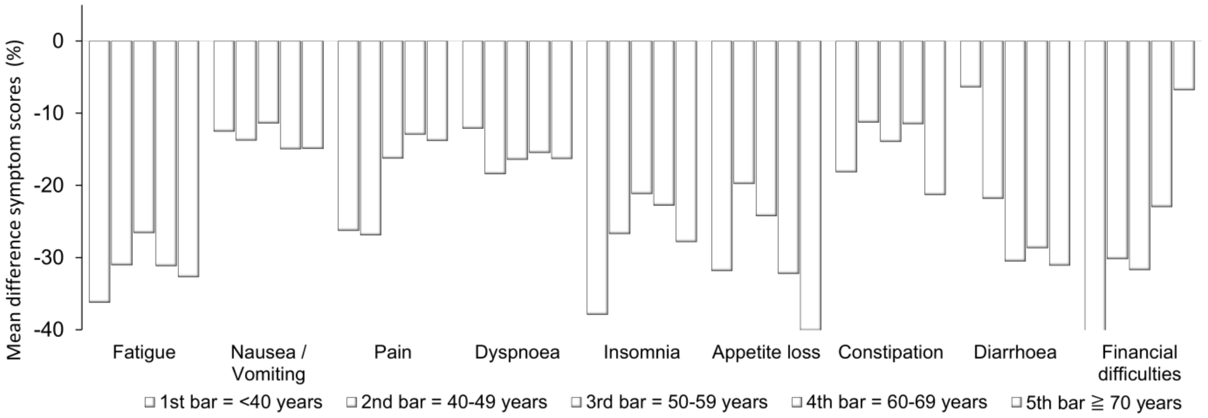
Mean difference symptom scores (%)
0
-10
-20
-30
-40
Fatigue
Nausea / Vomiting
Pain
Dyspnoea
Insomnia
Appetite loss
Constipation
Diarrhoea
Financial difficulties
1st bar = <40 years
2nd bar = 40-49 years
3rd bar = 50-59 years
4th bar = 60-69 years
5th bar ≧ 70 years

Supplementary figure 8: RCT-patients compared to the GGP1 surveyed at the time before RCT; functional scores (a), symptom scores (b), differences functional scores (c), differences symptom scores (d).

(a)

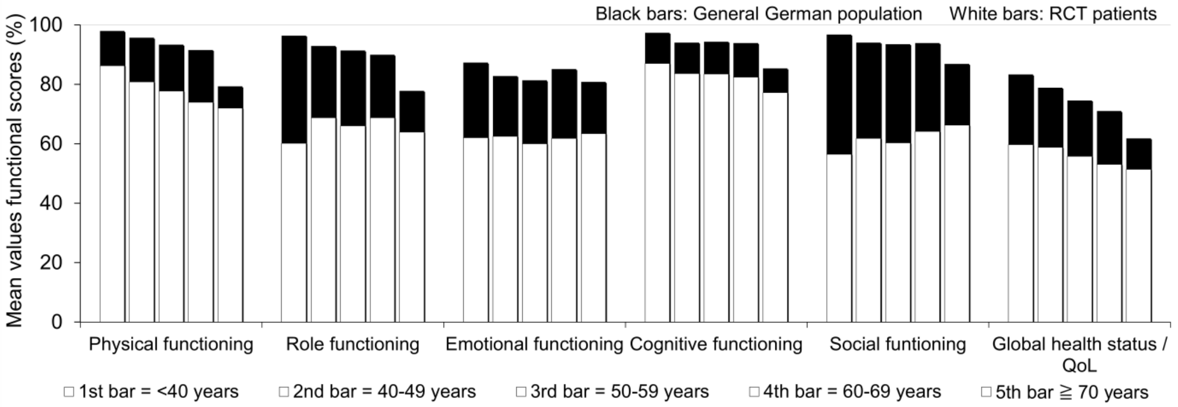

(b)

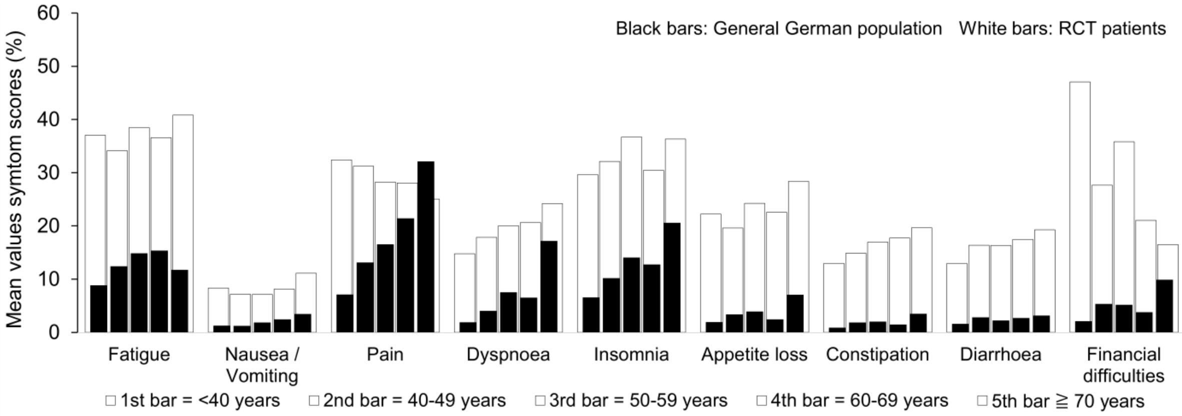

(c)

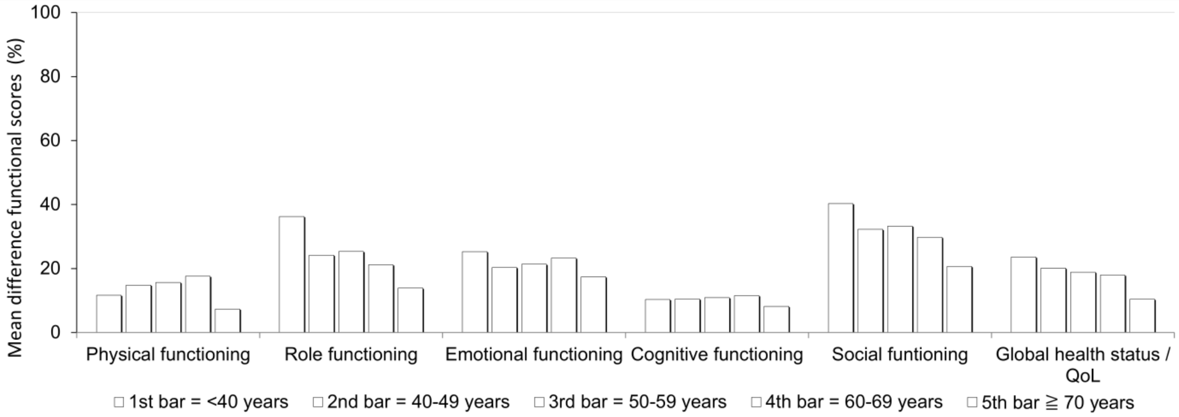

(d)

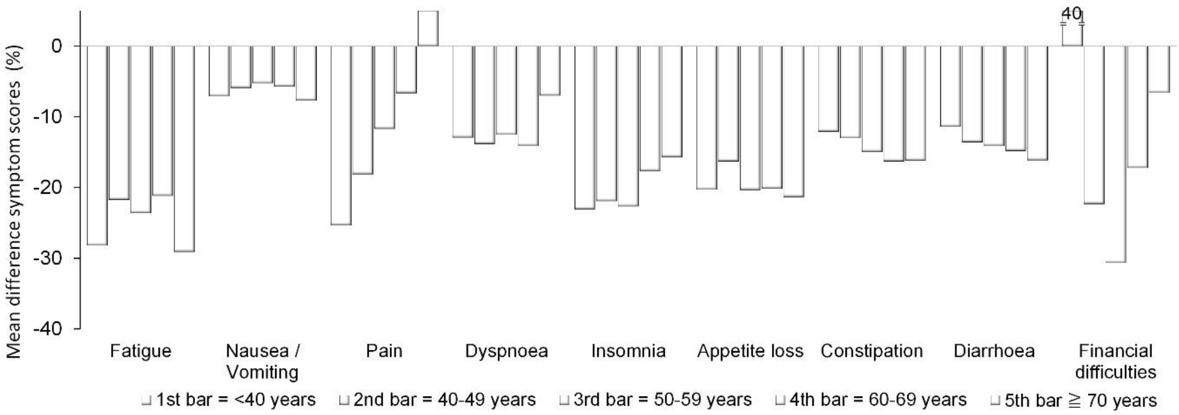

Mean difference symptom scores (%)
0
-10
-20
-30
-40
40
Fatigue
Nausea / Vomiting
Pain
Dyspnoea
Insomnia
Appetite loss
Constipation
Diarrhoea
Financial difficulties
1st bar = <40 years
2nd bar = 40-49 years
3rd bar = 50-59 years
4th bar = 60-69 years
5th bar ≧ 70 years

Supplementary figure 9: RCT-patients compared to the GGP1 surveyed at the time of the last week of RCT (6th week); functional scores (a), symptom scores (b), differences functional scores (c), differences symptom scores (d).

(a)

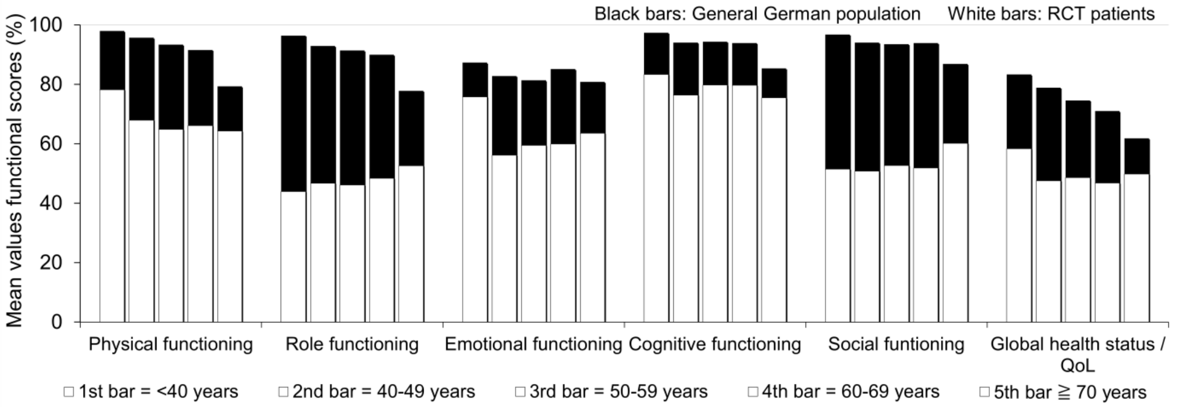

(b)

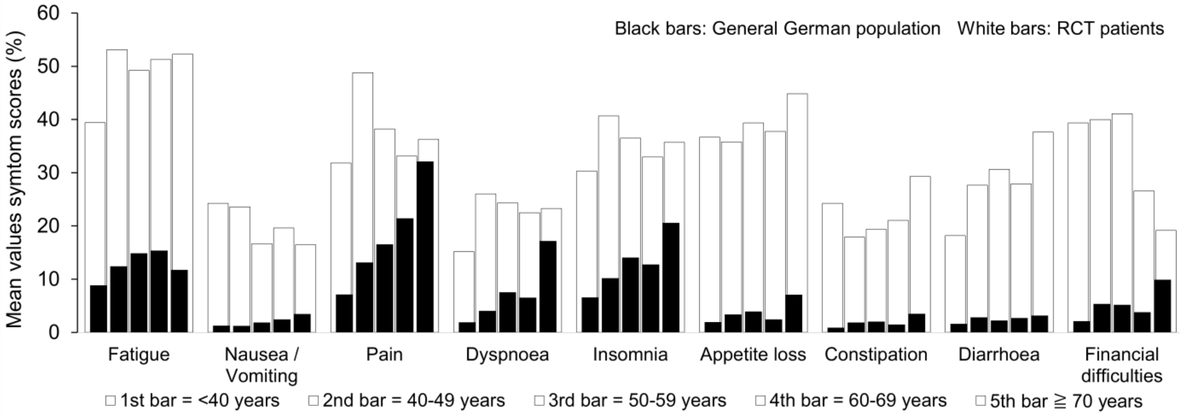

(c)

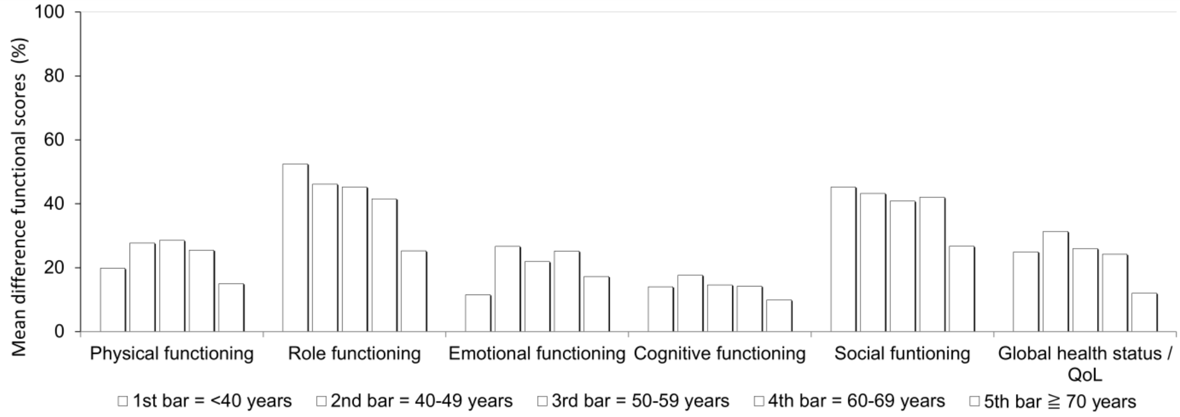

(d)

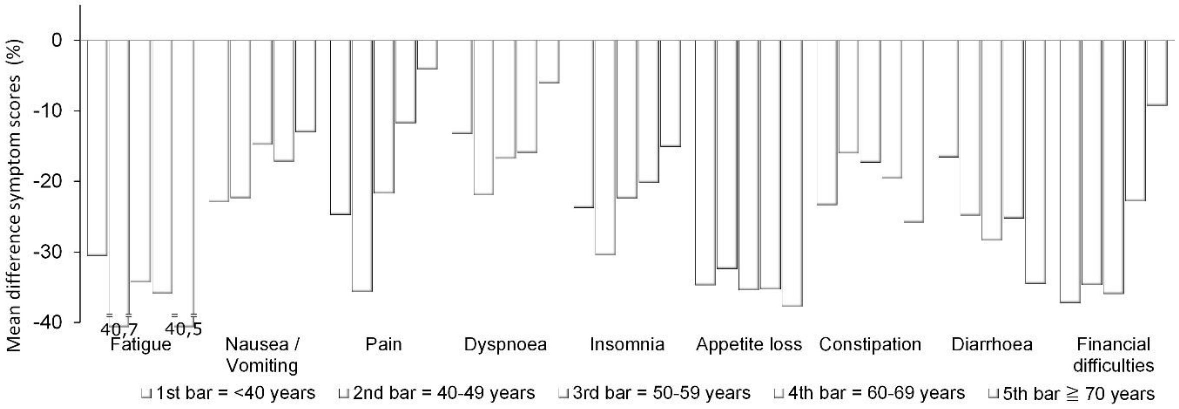
0
-10
-20
-30
-40
Mean difference symptom scores (%)
40,7
40,5
Fatigue
Nausea / Vomiting
Pain
Dyspnoea
Insomnia
Appetite loss
Constipation
Diarrhoea
Financial difficulties
1st bar = <40 years
2nd bar = 40-49 years
3rd bar = 50-59 years
4th bar = 60-69 years
5th bar ≧ 70 years

Supplementary figure 10: RCT-patients compared to the GGP1 surveyed at the time of 4-6 weeks after the end of RCT; functional scores (a), symptom scores (b), differences functional scores (c), differences symptom scores (d)

(a)

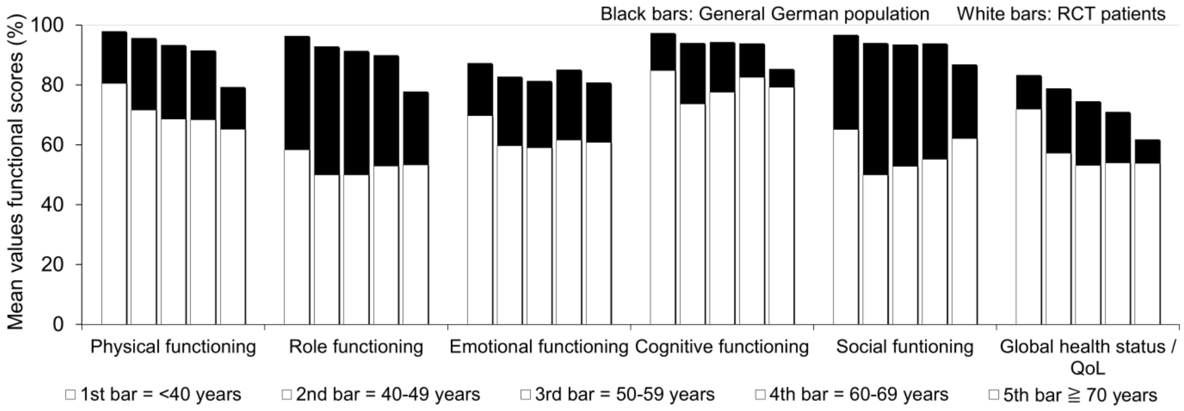

(b)

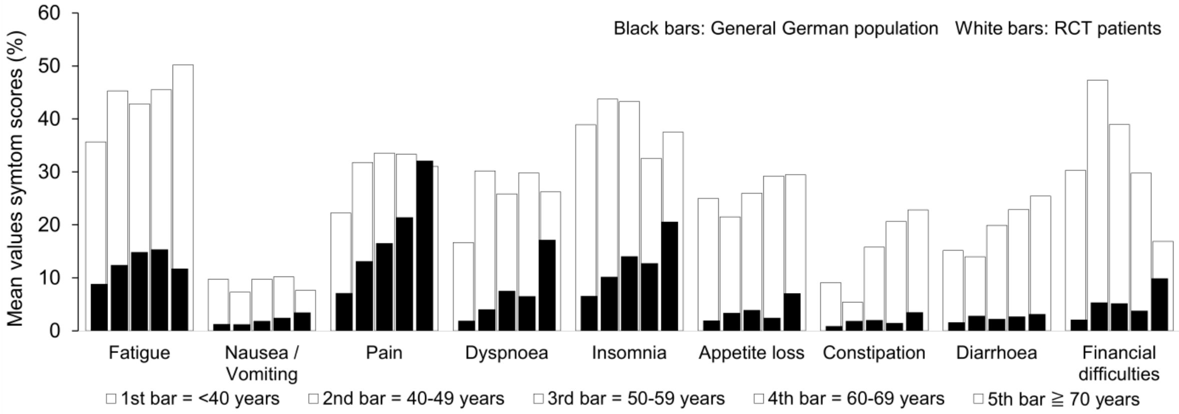

(c)

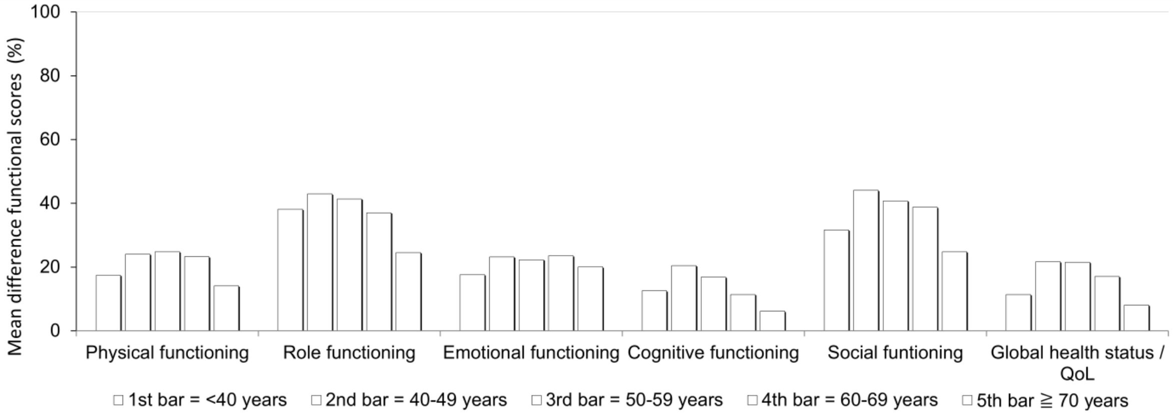

(d)

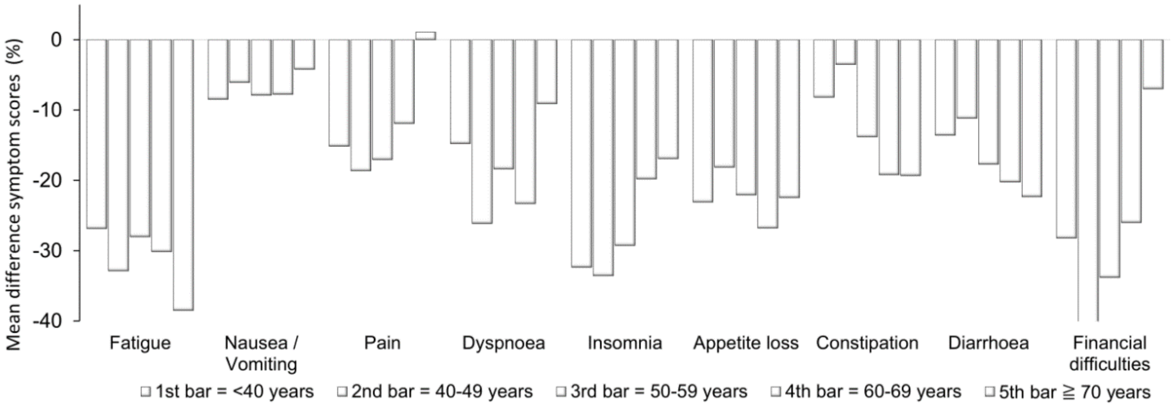
Mean difference symptom scores (%)
0
-10
-20
-30
-40
Fatigue
Nausea / Vomiting
Pain
Dyspnoea
Insomnia
Appetite loss
Constipation
Diarrhoea
Financial difficulties
1st bar = <40 years
2nd bar = 40-49 years
3rd bar = 50-59 years
4th bar = 60-69 years
5th bar ≧ 70 years

Supplementary figure 11: RCT-patients compared to the GGP1 surveyed at the time of the 1 year follow-up; functional scores (a), symptom scores (b), differences functional scores (c), differences symptom scores (d).

(a)

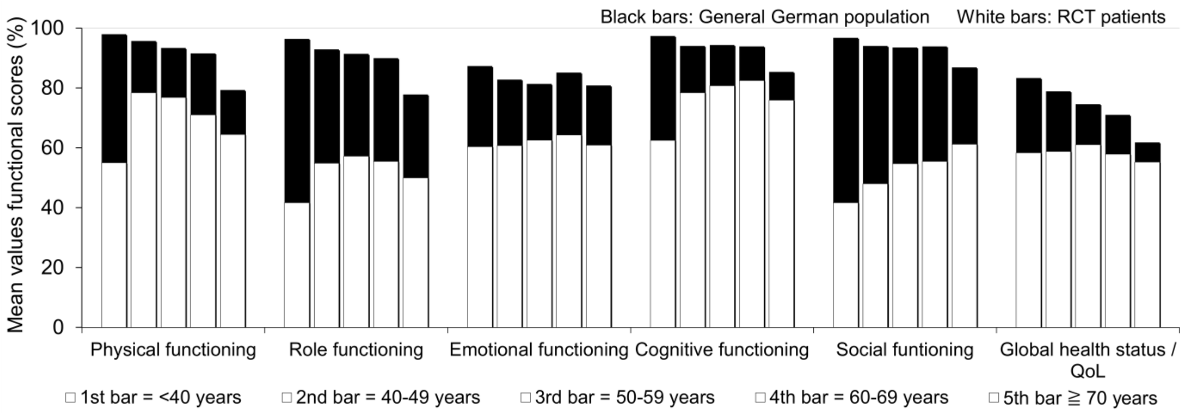

(b)

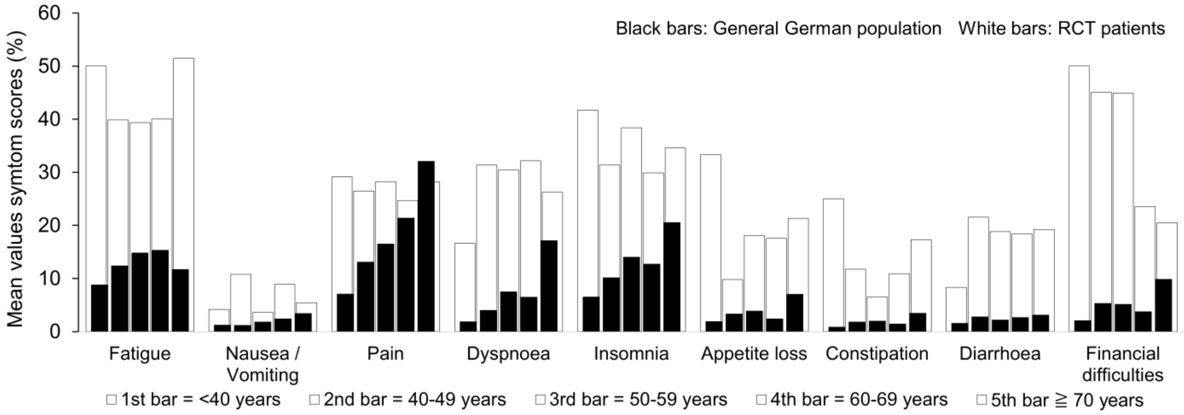

(c)

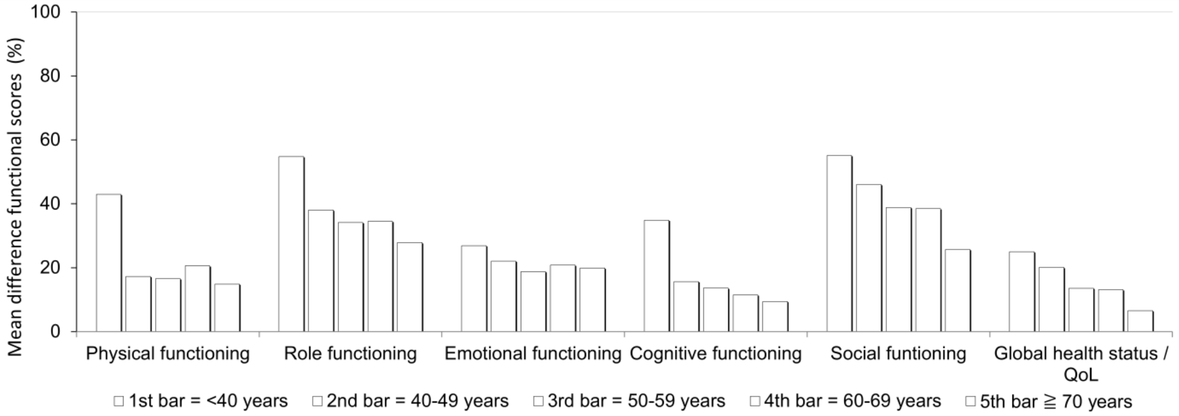

(d)

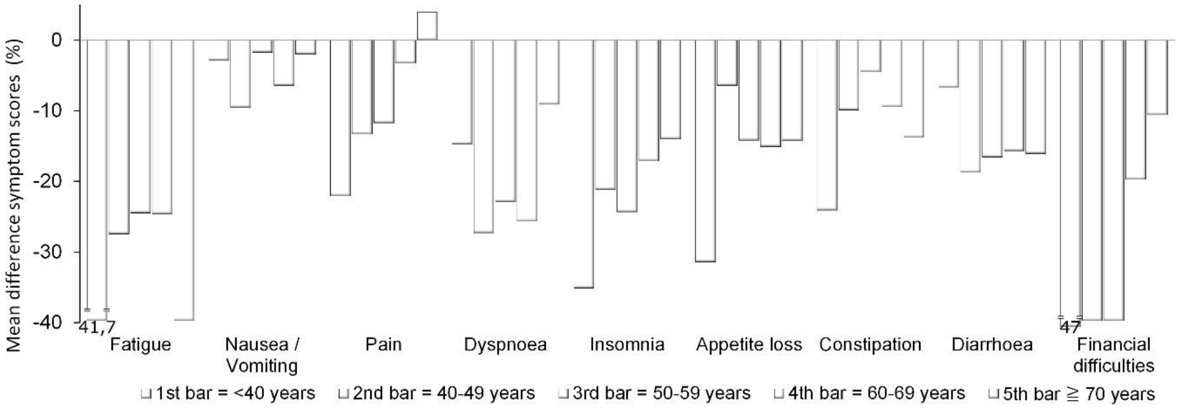
Mean difference symptom scores (%)
0
-10
-20
-30
-40
41,7
47
Fatigue
Nausea / Vomiting
Pain
Dyspnoea
Insomnia
Appetite loss
Constipation
Diarrhoea
Financial difficulties
1st bar = <40 years
2nd bar = 40-49 years
3rd bar = 50-59 years
4th bar = 60-69 years
5th bar ≧ 70 years

Supplementary table 1: Difference in percent points between GGP 1 (Hinz) and various subgroups and functional and symptom scores.

		age category						age category				
subgroup	score	< 40	40-49	50-59	60-69	> 70	score	< 40	40-49	50-59	60-69	> 70
GGP 2	physical functioning	3.6	5.2	6.6	9.0	4.6	cognitive functioning	*10.3*	*10.2*	*11.5*	8.4	6.8
men		3.9	1.6	0.3	0.5	2.5		3.1	2.4	0.7	0.7	1.7
women		6.7	1.7	0.6	1.6	9.2		4.7	2.5	1.4	2.4	5.7
inquiry period 1		6.2	6.5	5.2	2.8	4.7		3.0	6.0	3.4	0.5	0.2
inquiry period 2		2.0	6.4	7.7	5.1	3.0		0.7	1.3	0.3	3.1	2.0
inquiry period 3		0.4	2.7	3.9	2.8	2.0		0.8	4.0	2.5	0.3	1.8
inquiry period 4		*25.1*	4.1	4.3	0.2	2.8		*21.5*	0.8	0.8	0.4	1.5
GGP 2	role functioning	7.9	*10.7*	*12.7*	*11.7*	7.4	social functioning	7.7	*11.4*	*12.1*	*12.7*	9.7
men		4.6	0.4	0.4	0.0	3.9		4.5	0.9	3.1	2.0	1.5
women		7.6	0.5	0.9	0.0	*13.3*		6.5	0.9	6.4	6.9	5.3
inquiry period 1		6.6	*13.5*	*10.2*	*10.2*	7.3		0.5	8.5	4.9	6.1	3.4
inquiry period 2		9.7	8.5	9.8	*10.2*	4.1		4.5	2.5	2.7	6.2	2.6
inquiry period 3		4.7	5.3	5.9	5.6	3.3		9.1	3.3	2.5	2.9	0.7
inquiry period 4		*11.9*	0.4	1.4	3.1	6.6		*14.3*	5.3	0.7	2.6	1.6
GGP 2	emotional functioning	*16.7*	*16.7*	*14.0*	*10.1*	6.2	global health status / QoL	9.9	*12.2*	*10.1*	5.2	0.2
men		2.1	4.2	1.2	3.1	2.8		4.5	0.8	1.8	0.7	0.8
women		1.6	4.4	2.4	*10.5*	9.8		8.6	0.9	3.8	2.3	2.9
inquiry period 1		5.3	2.2	0.6	0.7	0.6		2.1	4.1	2.0	0.4	1.4
inquiry period 2		8.4	4.1	0.0	2.6	0.8		3.5	7.2	5.1	6.7	3.0
inquiry period 3		2.4	0.6	0.4	0.9	2.0		*10.1*	2.5	0.6	0.5	1.0
inquiry period 4		6.9	0.5	3.1	1.8	1.8		3.5	4.1	7.3	4.4	2.5
GGP 2	fatigue	*17.9*	*17.4*	*16.2*	*12.4*	8.1	insomnia	*12.1*	*15.5*	*17.8*	*15.0*	7.7
men		5.1	0.0	0.8	1.3	4.2		6.0	1.3	1.7	1.3	3.5
women		7.1	0.0	1.7	4.4	*14.2*		*11.1*	1.4	3.4	4.4	*12.2*
inquiry period 1		0.9	9.3	4.6	5.5	*10.6*		3.7	6.1	1.9	0.7	0.1
inquiry period 2		1.5	9.7	6.1	9.2	*22.1*		3.0	2.4	2.1	1.8	0.6
inquiry period 3		2.2	1.9	0.2	3.4	*20.0*		5.6	5.5	4.7	1.4	1.2
inquiry period 4		*12.1*	3.6	3.7	2.1	*21.3*		8.4	6.9	0.2	1.3	1.6
GGP 2	nausea/ vomiting	2.7	3.0	2.6	0.1	0.2	appetite loss	5.4	4.4	4.7	4.8	1.6
men		1.5	2.5	1.4	1.9	2.3		4.8	0.5	0.6	2.3	4.7
women		1.7	2.6	2.9	6.6	7.7		7.8	0.6	1.2	8.2	15.8
inquiry period 1		3.7	5.2	3.2	2.6	0.5		3.7	4.0	2.7	3.9	3.0

inquiry period 2		*12.2*	*11.2*	6.4	8.9	5.8		*10.7*	*12.1*	*12.4*	*11.3*	*13.5*
inquiry period 3		2.3	5.1	0.6	0.5	3.0		0.9	2.2	1.0	2.7	1.8
inquiry period 4		7.9	1.6	6.7	1.8	5.2		7.4	*13.9*	8.9	8.9	*10.0*
GGP 2	pain	*10.8*	*12.0*	*14.0*	6.3	1.7	constipation	2.4	3.3	4.3	4.6	8.9
men		2.9	3.8	0.2	1.5	4.6		2.6	0.2	0.5	1.2	0.7
women		4.6	3.9	0.5	4.9	16.3		5.1	0.2	1.1	4.2	2.6
inquiry period 1		3.7	4.8	4.1	1.3	4.6		1.0	1.6	0.0	0.6	2.5
inquiry period 2		3.2	*12.7*	5.9	3.7	6.6		*10.3*	4.6	2.4	4.0	7.1
inquiry period 3		6.5	4.3	1.2	3.9	1.4		4.9	7.9	1.2	3.5	0.6
inquiry period 4		0.5	9.6	4.0	4.7	1.4		*11.0*	1.5	*10.5*	6.2	4.9
GGP 2	dyspnoea	4.6	7.6	8.1	7.4	0.1	diarrhoea	7.4	8.2	7.1	5.5	5.8
men		0.1	2.9	0.2	0.1	4.8		3.3	2.3	3.5	1.9	1.5
women		0.3	3.0	0.4	0.3	*11.6*		6.7	2.5	7.6	6.5	5.4
inquiry period 1		0.5	7.6	4.3	1.0	2.4		1.6	5.7	8.7	7.3	9.5
inquiry period 2		0.8	0.5	0.0	0.8	1.5		3.6	5.6	5.5	3.1	8.9
inquiry period 3		2.3	4.7	1.5	8.2	4.4		0.6	8.1	5.2	1.9	3.4
inquiry period 4		2.3	5.9	6.1	*10.5*	4.4		6.3	0.5	6.3	6.4	9.6
GGP 2	financial difficulties	4.7	6.4	9.9	*11.2*	6.0						
men		5.0	1.2	1.3	0.7	0.4						
women		8.6	1.4	2.8	2.4	1.5						
inquiry period 1		7.9	9.2	3.8	3.2	1.6						
inquiry period 2		0.2	3.2	1.5	2.3	1.0						
inquiry period 3		8.9	*10.5*	0.7	5.5	1.3						
inquiry period 4		*10.8*	8.3	5.3	0.7	2.4						
